# Flexible Distributed Lag Models for Count Data Using mgcv

**DOI:** 10.1080/00031305.2025.2505514

**Published:** 2025-07-03

**Authors:** Theo Economou, Daphne Parliari, Aurelio Tobias, Laura Dawkins, Hamish Steptoe, Christophe Sarran, Oliver Stoner, Rachel Lowe, Jos Lelieveld

**Affiliations:** a Department of Mathematics and Statistics, University of Exeter, Exeter, UK; b Climate and Atmosphere Research, The Cyprus Institute, Nicosia, Cyprus; c Laboratory of Atmospheric Physics, Aristotle University of Thessaloniki, Thessaloniki, Greece; d Institute for Environmental Assessment and Water Research, Spanish National Research Council, Barcelona, Spain; e Met Office, Exeter, UK; f School of Mathematics and Statistics, University of Glasgow, Glasgow, UK; g Barcelona Supercomputing Center (BSC), Barcelona, Spain; h Catalan Institution for Research and Advanced Studies (ICREA), Barcelona, Spain; i London School of Hygiene & Tropical Medicine, London, UK; j Max Planck Institute for Chemistry, Mainz, Germany

**Keywords:** Bayesian inference, DLNM, Environmental epidemiology, Heat-stress, Penalized splines

## Abstract

In this tutorial we present the use of R package mgcv to implement Distributed Lag Non-Linear Models (DLNMs) in a flexible way. Interpretation of smoothing splines as random quantities enables approximate Bayesian inference, which in turn allows uncertainty quantification and comprehensive model checking. We illustrate various modeling situations using open-access epidemiological data in conjunction with simulation experiments. We demonstrate the inclusion of temporal structures and the use of mixture distributions to allow for extreme outliers. Moreover, we demonstrate interactions of the temporal lagged structures with other covariates with different lagged periods for different covariates. Spatial structures are also demonstrated, including smooth spatial variability and Markov random fields, in addition to hierarchical formulations to allow for non-structured dependency. Posterior predictive simulation is used to ensure models verify well against the data.

## 1. Introduction

Distributed lag models (DLMs) are well-established tools for capturing the temporally lagged effects of a covariates. First introduced in econometrics (Almon 1965), they have now become the de facto modeling tool in epidemiological studies (e.g., Lubczyńska, Christophi, and Lelieveld 2015; Royé, Íñiguez, and Tobías 2020; Parliari et al. 2024), for linking environmental covariates with health outcomes (e.g., temperature with mortality). For counts $y_{t}$ (e.g., mortality) and covariate $x_{t}$ (e.g., temperature) a DLM can be generically defined as

(1) $$y_{t}∼\text{Poisson}(\mu_{t})$$

(2) $$\;\text{log}\;(\mu_{t})=\alpha +\beta_{0}x_{t}+\beta_{1}x_{t-1}+\cdots +\beta_{L}x_{t-L}.$$

The mean count is $\mu_{t}$ and *L* is the maximum number of lags. This is a Generalized Linear Model (GLM) that is likely to be unstable due to high correlation among $x_{t-l}$. Constraining the coefficients $\beta_{l}$, for example $\beta_{l}=g(l)$ where *g* is some unknown smooth function is one way to remedy this issue. Other ways of constraining $\beta_{l}$ include methods that assume they are random and jointly distributed (Welty et al. 2009; Obermeier et al. 2015).

DLMs have been extended to distributed lag *non-linear* models (DLNMs) where the effect of $x_{t-l}$ at each *l* is nonlinear (Armstrong 2006; Gasparrini, Armstrong, and Kenward 2010). This greatly increases modeling flexibility so that the linear predictor in (2) becomes

(3) $$\;\text{log}\;(\mu_{t})=\alpha +h(0,x_{t})+h(1,x_{t-1})+\cdots +h(L,x_{t-L})$$

$$=\alpha +\sum_{l=0}^{L}h(l,x_{t-l})$$

where $h(l,x_{t-l})$ is a two dimensional smooth function. Smoothness in the lag dimension induces the necessary constraint.

A further consideration in using smooth functions in DLNMs is penalization. Too much flexibility in the unknown functions may result in over-fitting the data (Wood 2017). Zanobetti (2000) first used penalized Generalized Additive Models (GAMs) to deal with the issue, while later on, Obermeier et al. (2015) used GAMs to penalize the lagged effects for *L* being too large. More generally, DLNMs using penalized splines were used in hydrology (Rushworth et al. 2013), while Wilson et al. (2017) used truncated basis functions in Bayesian setting to construct and penalize the lagged effects. A key reference on penalized DLNMs is Gasparrini et al. (2017), who present a thorough exposition of using GAMs to fit DNLMs, including a discussion on the type of penalties, splines, estimation procedures and their relative merits. Moreover, recent work (Bender et al. 2019) presents flexible and penalized DLNMs by fitting them as Generalized Additive Mixed Models, a viewpoint we adopt here. We note however that other ways exist to fit penalized DLNMs, for example using Gaussian Process regression (Heaton and Peng 2012, 2014)

A further important extension to DLNMs is the interaction of the covariate-lag relationship with other factors such as other environmental variables or indeed other dimensions such as space, season etc. For example, Muggeo (2007) propose a model with an interaction between temperature, lag and air-quality, whereas Chien et al. (2018) fit a model that interacts the covariate-lag function with a spatial Markov random field to allow for spatial structure in the relationship. Moreover, a two-stage approach (Dominici, Samet, and Zeger 2000; Gasparrini and Armstrong 2013) has been proposed to pool estimates from individual DLNMs across spatial regions using a meta-analytic framework.

In this tutorial we provide an exposition of fitting DLNMs using the R package mgcv (Wood 2011, 2017), which uses penalized regression splines to construct the smooth functions. We fit DLNMs as penalized GAMs, and show that many important modeling extensions to the “baseline” DLNM can be implemented using the capabilities of mgcv. We demonstrate the handling of interactions, spatial and temporal variability, the use of mixture models for extreme outliers, and also the formulation of hierarchies for data pooling across grouping structures. Using approximate simulation-based Bayesian inference facilitates interpretation, uncertainty quantification and model checking.

Section 2 presents penalized GAMs in mgcv and how they are used to fit DLNMs. Section 3 demonstrates the fitting of simple DLNMs using both simulated and real data, including implementation of temporal structures. Section 4.3 covers more complex models, including interactions, spatial structures and hierarchical formulations. Finally, Section 6 presents the summary, a discussion and some conclusions.

## 2. GAMs in Brief

In penalized GAMs, the effect of covariate *x* is represented by an unknown smooth function:

(4) $$f(x_{i})=\sum_{k=1}^{K}\beta_{k}b_{k}(x_{i})=X_{i}\beta$$

where $b_{k}$ are basis functions, $\beta_{k}$ are coefficients and X is the model matrix. Function f(·) is penalized when *K* (the number of coefficients) is too large, which would result in f(·) being too “wiggly” and thus the model over-fitting the data. Inference is based on:

(5) $$l(\beta ,\phi ;y)-\lambda \int_{x}f^{\prime \prime }{\left(x\right)}^{2}\text{dx}=l(\beta ,\phi ;y)-\lambda \beta^{\prime }\mathrm{S\beta}$$

where l(·) is the log-likelihood, λ is a penalty parameter and S is a penalty matrix that relates to a quadratic penalty on β. Matrix S depends on the choice of basis functions as well as any constraints on the function. One such constraint is (by default in mgcv) that the function sums to zero over the observed covariate values, that is $\sum_{i}f(x_{i})=0$. This ensures identifiability of the intercept, particularly in the presence of other smooth functions. In practice, *K* is chosen to be larger than necessary while penalization via λ guards against over-fitting. The amount of penalization is estimated from the data as a compromise between out-of-sample and in-sample predictive skill (Wood 2017).

### 2.1. Bayesian Interpretation of GAMs

The smoothness of f(·) in (4) can be viewed as a constraint on β and from a Bayesian viewpoint, this can be represented with a prior distribution $\beta ∼N(0,S^{-}/\lambda )$, where $S^{-}$ is the pseudo-inverse of S. Assuming the coefficients are random, allows estimation via restricted maximum likelihood (REML). Given estimates of the penalty parameter and any dispersion parameter ϕ, the posterior distribution can be approximated (Wood 2017) by:

(6) $$\beta |y,\lambda ,\phi ∼N\left(\hat{\beta },{\left(X^{\prime }\text{WX}+\hat{\lambda }S\right)}^{-1}\hat{\phi }\right),$$

where W is the weight matrix associated with the penalized iterative re-weighted least squares algorithm used to fit GAMs (Wood 2017) and $\hat{\beta }$, $\hat{\phi }$, $\hat{\lambda }$ are the REML estimates. All components of (6) are readily provided when fitting GAMs in mgcv. The posterior predictive distribution (PPD) of any response value $\tilde{y}$ (given the data, λ and ϕ) is

(7) $$p(\tilde{y}|y,\lambda ,\phi )=\int_{\beta }p(\tilde{y}|\beta ,\lambda ,\phi )p(\beta |y,\lambda ,\phi )d\beta .$$

### 2.2. Interactions

Functions of multiple covariates can be constructed using tensor product smooths. A function of *x* and *z* say, is defined by letting the coefficients of (4) to be smooth functions of *z*:

(8) $$\begin{array}{cc} f(x_{i},z_{i}) & =\sum_{k=1}^{K}\beta_{k}(z_{i})b_{k}(x_{i})\;\text{ where }\\ \beta_{k}(z_{i}) & =\sum_{j=1}^{J}\gamma_{k,j}a_{j}(z_{i}) \end{array}$$

(9) $$\begin{array}{cc} \Rightarrow f(x_{i},z_{i}) & =\sum_{k=1}^{K}\sum_{j=1}^{J}\gamma_{k,j}a_{j}(z_{i})b_{k}(x_{i})=P_{i}\gamma \end{array}$$

where $\gamma_{k,j}$ are coefficients, a(·) are basis functions and P is the resulting (n×(J·K)) model matrix. Higher dimensional functions can be constructed in the same way at the expense of exponential growth in the number of coefficients.

### 2.3. DLNMs as GAMs in mgcv

DLNMs can be fitted in mgcv where $h(l,x_{t-l})$ in (3) is constructed as a tensor product and implemented using the linear.functional.terms option. Smoothness and penalization of the tensor products ensures optimal use of the data (see also Gasparrini et al. 2017).

### 2.4. Interpretation and Inference

In the epidemiological context, the (log) mean count given a single covariate $x_{t}$ is

(10) $$\;\text{log}\;(\mu_{t})=\alpha +\sum_{l=0}^{L}h(l,x_{t-l})+\;\text{log}\;(O_{t}).$$

Function $h(l,x_{t-l})$ in mgcv is by default constrained to sum-to-zero over the observed covariate values, so it is the additive change in the overall log mean count or, in the presence of an offset $O_{t}$ (e.g., population), it is the change in the mean occurrence rate per unit population. Equivalently, $\text{RR}(l,x_{t-l})=\;\text{exp}\;\{h(l,x_{t-l})\}$ can be interpreted as the relative risk (RR), the multiplicative change with respect to the overall mean count or rate  exp {α}. Sometimes it is desirable to compute the RR in relation to mortality at a specific value of the covariate say $\tilde{x}$, which can be computed by $\text{RR}(l,x_{t-l})=$ $\;\text{exp}\;\{h(l,x_{t-l})-h(l,\tilde{x})\}$.

**Figure 1:**
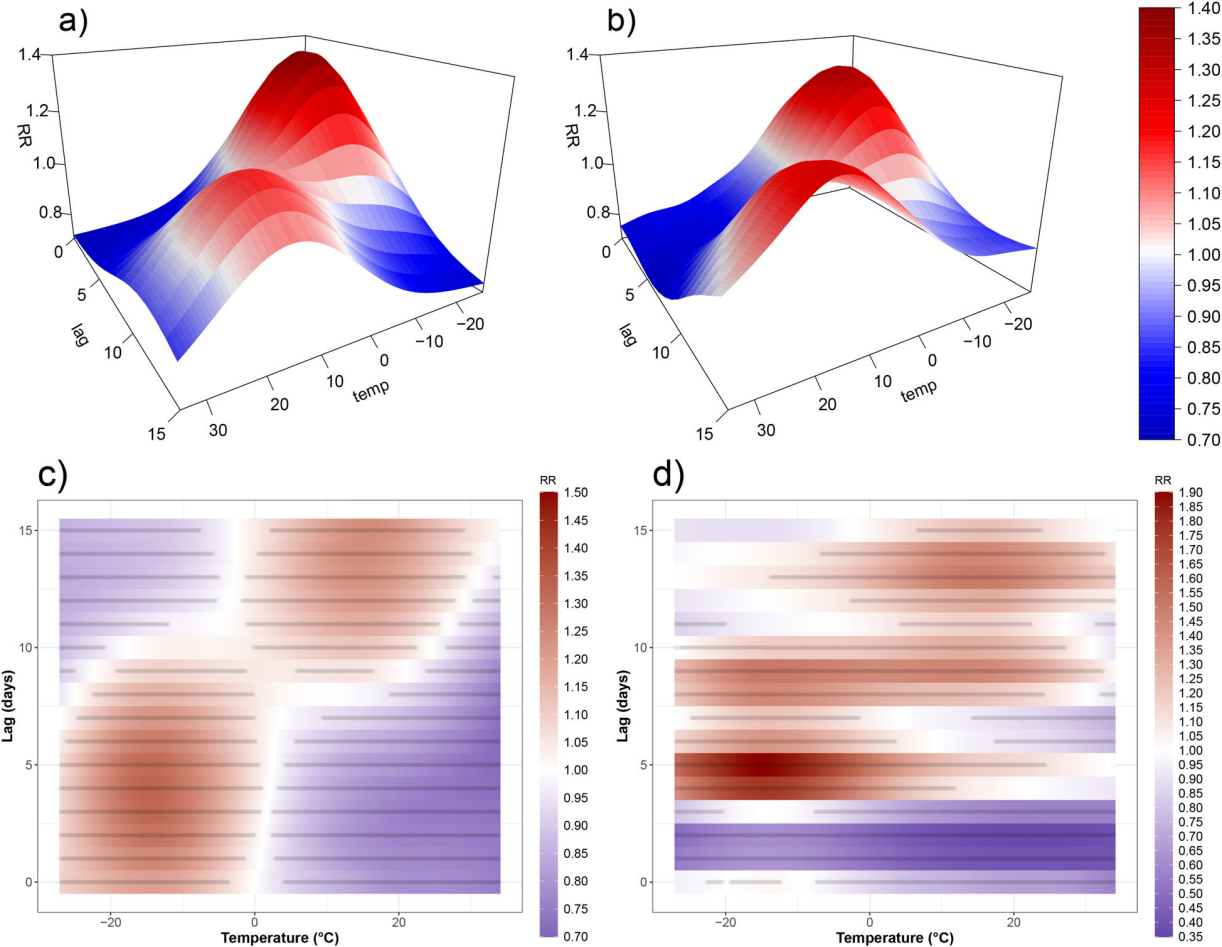
Relative risk estimates for the simulated data. Red color indicates RR values >1 while blue color signifies RR values <1. Panel a) shows the “true” RR surface; panel b) shows the estimated RR surface from a Poisson GAM; panel c) shows the same as b) but as a raster plot with a gray point for each grid point where the RR is significantly different from 1; and d) shows the RR estimate from an un-penalized Poisson GAM.

Conventionally, effects are illustrated using a plot of RR(l,x) over a grid of finite values for *x* and *l* as in Figure 1(b). Equivalently, the same information can be shown as 2D raster plots as in Figure 1(c). Such plots are counterfactual statements: $x_{t-l}$ is fixed for all lags *l* (e.g., $x_{t-l}=40^{{}^{\circ}}$C for L=20 days) and we see the RR associated with each lag. An often used summary of these effects is the “cumulative risk” (CR) defined as

(11) $$\text{CR}(x_{t})=\;\text{exp}\;\left\{\sum_{l=0}^{L}h(l,x_{t-l})\right\}$$

which quantifies the total risk when $x_{t}$ is the same for all *l*.

Function *h* depends on the coefficients so inference on functions of *h* (RR and CR) can be performed using Monte Carlo simulation. Checking whether the value 1 lies inside a 95% (posterior) credible interval for RR(l,x) indicates strong weight of evidence that the RR is not significantly different from the baseline. Moreover, predictions of the counts $y_{t}$ (7) enables comprehensive model checking (Gelman et al. (2013), chap. 6).

In what follows, we demonstrate various modeling options to simulated and real data, focusing on flexibility, interpretability and model checking.

## 3. Basic DLNMs

### 3.1. Simulated Data

Figure 1(a) shows a fictitious $\text{RR}(l,x_{t-l})$ surface of temperature and lag. A real timeseries of daily temperature (1987–2000) in Chicago (Tobias and Madaniyazi 2024) was used to simulate Poisson counts with L=15 days. A Poisson GAM with $\;\text{log}\;(\mu_{t})=\alpha +\sum_{l=0}^{15}h(l,x_{t-l})$ was fitted to the simulated data via:

> gam(y∼te(TEMP,LAG,k = c(10,10), bs = c("tp","tp")),

family = poisson,data = simDat)where te() constructs h(l,x) as a tensor product of two marginal thin-plate regression splines (TPRS), each with 10 knots. The TPRS basis is an attractive choice as it avoids explicit knot placement (Wood 2017), but see also (Gasparrini et al. 2017) for details on choosing spline bases for DLNMs. The function has $10^{2}-1=99$ coefficients (the minus one is for the sum-to-zero constraint). Figure 1(b) shows the resulting RR estimate, which is a close match to the true RR. Figure 1(c) shows the same estimate as a raster plot, along with gray points for any RR value whose credible interval does not contain 1. The intervals were constructed using 1000 Monte Carlo samples (see (6) and the online code).

The function k.check() checks whether the number of coefficients is adequate:

> k.check(model)

k’ edf

te(TEMP,LAG) 99 63.8107

After penalization, the effective degrees of freedom (edf) is ≈64 suggesting that 99 (>64) coefficients are enough. The same model without penalization results in the estimate shown in Figure 1(d) (note the different color scale), which is very different to the true RR surface. This simple example illustrates the importance of objective penalization, noting in particular that even spurious relationships may appear “significant” with enough data, for example, bottom left corner of Figure 1(d).

We quantify characteristics of the estimates via the differences between the true RR values (Figure 1(a)) and the estimates (Figure 1(b)), in each of 10 equidistant quantiles of temperature. We use the raw mean differences (the bias) and the root mean squared differences (the variability) to quantify the accuracy of the estimates. The supplementary material shows a boxplot of the bias, indicating that the estimates are unbiased (zero of average) across temperature quantiles. The values range from –0.05 to 0.05 (RR scale) with no visible pattern across quantiles. Similarly, there is no discernible trend in the variability of the estimates, which is centered at 0.05.

### 3.2. Temporal Structures, Model Checking and Model Expansion

**Figure 2:**
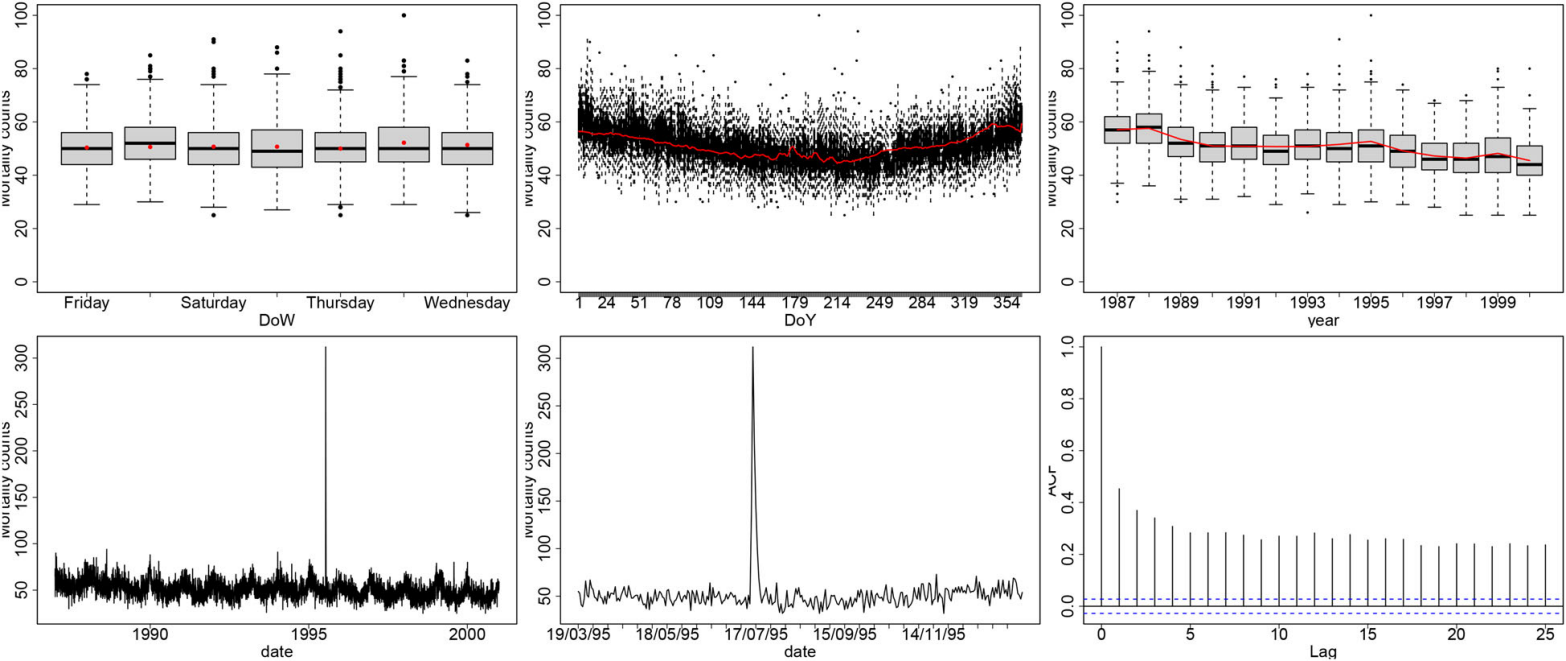
Chicago mortality. Row-wise from top left to bottom right: day-of-week boxplot; day-of-year boxplot; yearly boxplot; count timeseries; count timeseries for March–December 1995; Autocorrelation plot. In red, we show the means of posterior predictive samples.

We proceed with real data: daily mortality for Chicago during 1987–2000, as well as the recorded daily temperature. Exploratory analysis of the count timeseries (Figure 2) indicates a strong seasonal cycle; a weak day-of-week effect; 5 very large and consecutive outliers; a smooth decreasing trend across the 14 years; and low but fairly weak autocorrelation. We fit a Negative Binomial (NB) GAM:

(12) $$y_{t}∼\text{NB}(\mu_{t},\theta )$$

(13) $$\;\text{log}\;(\mu_{t})=\alpha +\sum_{l=0}^{L}h(l,x_{t-l})+\gamma_{\text{dow}(t)}$$

$$+f_{1}(\text{doy}(t))+f_{2}(\text{year}(t)),$$

where θ is the dispersion hyperparameter and year(t) denotes the calendar year that *t* occurs in. $\gamma_{\text{dow}(t)}$ is a day-of-week random effect, $f_{1}$ is a smooth function of day-of-year (constructed using a cyclic cubic regression spline) and $f_{2}$ is a smooth function of year (constructed using a TPRS). Choosing L=20 is common when studying temperature effects on mortality (Tobías, Armstrong, and Gasparrini 2017; Royé, Íñiguez, and Tobías 2020). Unlike the Poisson, the NB distribution automatically deals with overdispersion, a very common issue in count data. The model is fitted via:

> model1 < - gam(y∼te(TEMP,LAG,k = c(10,10),

bs = c("tp","tp")) + s(DoW,bs="re") + s(DoY,bs="cc",k = 30) + s(year,k = 9),

family = nb,knots = list(DoY = c(0,366)))

To assess model1 we use posterior predictive model checking (Gelman et al. 2013). As discussed in Section 2.4, we produce posterior predictive samples corresponding to each observed count. We plot the posterior predictive mean count for each of: day-of-week, day-of-year and year in Figure 2 (prediction intervals not shown for clarity). The estimates appear reasonable, although the seasonal cycle may not be the same in each year.

Table 1 shows a comparison of the sample mean, variance, interquartile range, and the 0.01 and 0.99 quantiles of the observed mortality counts, with corresponding estimates from model1. Clearly, the sample variance is underestimated.

**Table 1:** Summary statistics of the Chicago mortality date and corresponding posterior predictive means from model1 and model2.

Statistic	Observations	model1	model2
		(35340)	(34793)
Mean	50.85	50.85 [50.54, 51.16]	50.85 [50.54, 51.15]
Variance	107.52	99.36 [94.84, 104.21]	111.68 [89.42, 160.66]
IQR	13	13.06 [13.00, 14.00]	12.87 [12, 13]
1st quantile	32	30.48 [30, 31]	31.36 [31, 32]
99th quantile	75	76.80 [75, 78]	74.37 [73, 76]

NOTE: 95% prediction intervals are given in square brackets. The number under each model name is the AIC.

**Figure 3:**
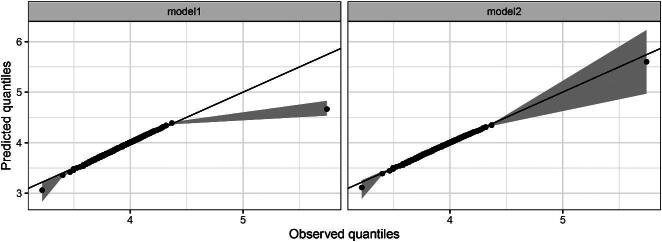
Predicted versus observed quantiles at the log-scale, for the 4 models applied to the Chicago data.

We also compute a sequence of quantiles for the observations and the predictions to better compare their overall distributions. Using 200 equidistant quantiles (between 0 and 1, both included), Figure 3 indicates poor agreement particularly for the maximum observed count of 312 (one of the five extreme outliers).

**Figure 4:**
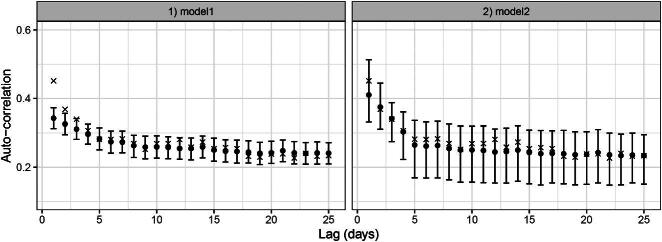
Autocorrelation plots of the predictions from model1 and model2. The “x” symbols are the observed autocorrelation values; the points are the posterior mean autocorrelations, while whiskers represent the 95% prediction intervals.

Moreover, Figure 4 shows the autocorrelation of the observed counts relative to the predictions. For model1, the autocorrelation is under-estimated for lags 1 and 2.

To improve auto-correlation and outlier estimation we expand the model using the exploratory analysis. First we allow the day-of-year effect to be different for each year. Also, removing the five extreme outliers greatly improves model1 but rather than waste potentially important data we allow the five outliers to follow a different NB distribution with its own intercept and dispersion parameter, but with all other components being shared. Let ω(t)=1 when the count y(t) is not one of the five outliers and ω(t)=2 otherwise. The model is

(14) $$y_{t}∼\text{NB}\left(\mu_{t},\theta_{\omega (t)}\right)$$

(15) $$\;\text{log}\;(\mu_{t})=\alpha_{\omega (t)}+\sum_{l=0}^{L}h(l,x_{t-l})+\gamma_{\text{dow}(t)}$$

$$+\sum_{j=1987}^{2000}I(j=\text{year}(t))\beta_{\text{year}(t)}+f_{\text{year}(t)}(\text{doy}(t)).$$

where I(c)=1 if condition *c* is true and zero otherwise and $\beta_{\text{year}(t)}$ are fixed effect coefficients for each year. This mixture model allows the outliers to inform estimation of the smooth functions, while recognizing that they may have different overall mean and dispersion (Economou et al. 2023; Economou, Johnson, and Dyson 2024). The model is fit using the gfam family in mgcv: model2 < - gam(Response∼omega + te(TEMP,LAG, k = c(10,10),bs = c("tp","tp")) + fYear + s(DoY,bs="cc",k = 30, by = fYear) + s(DoW,bs="re"), family = gfam(list(nb,nb)),

knots = list(DoY = c(0,366)))where s(DoY,bs="cc",k = 30,by = fYear) gives a different cyclic smooth function per year, and fYear is a factor with one level for each year (in place of a temporal trend). Note that two Negative Binomial distributions are specified in the family argument, to ensure that a different dispersion parameter ($\theta_{w(t)}$) is estimated for each the outlier and non-outlier cases.

The overall mean count for the non-outliers (in 1987) was estimated as 61.06, as opposed to 205.67 for the outliers, while ${\hat{\theta }}_{1}=793.79$ compared to ${\hat{\theta }}_{2}=5.43$, indicates much higher variability in the outliers. The model is a very good fit to the data in terms of (a) the QQ plot in Figure 3, (b) the autocorrelation plot in Figure 4 and (c) the summary statistics in Table 1. The Akaike Information Criterion (AIC), an estimate of out-of-sample prediction error (Gelman et al. 2013) confirms that model2 is much better than model1 (Table 1). We thus proceed to interpret the results from model2.

### 3.3. RR, CR, and Attributable Fraction

**Figure 5:**
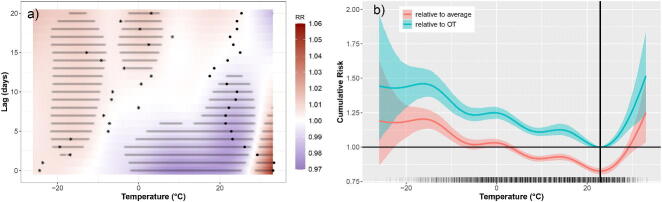
Risk estimates for model2. Panel a) shows RR estimates with regions of significant difference from RR =1 (at 95% level) shaded in gray. Black points show the temporal trajectory of a hot day (July 14, 1995) while stars indicate the trajectory of a cold day (February 03, 1996). Panel b) shows cumulative risk in red, and also in blue for when the baseline risk reflects the OT (vertical line). The rug relates to the number of observations.

Figure 5(a) shows the estimated RR from model2. There is significant elevated risk (≈5% above average) for very high temperature values ($>30^{{}^{\circ}}$C) for up to 7 lags (e.g., heat-waves). Mildly elevated risk at low temperatures at longer lags is evident, likely due to transmission of communicable diseases such as influenza (Barreca 2012; Zeng et al. 2017). A mortality “sweet-spot” appears in 0–$22^{{}^{\circ}}$ over lags 5–10.

Figure 5(b) shows the cumulative risk CR(x) (11), which is elevated for $>30^{{}^{\circ}}$C and $<10^{{}^{\circ}}$C, while mortality is below average in 3${}^{{}^{\circ}}$C–27${}^{{}^{\circ}}$C. The temperature value corresponding to the minimum CR(x) was termed the “optimum temperature” or OT (Honda et al. 2014), here approximated at 22.9${}^{{}^{\circ}}$C. Figure 5(b) shows the CR when the baseline risk is the mortality rate at the OT (Section 2.4).

Both the *RR* and the *CR* are counterfactual. For instance, a vertical “slice” along the lag dimension in Figure 5(a) implies that temperature is fixed for 21 days (an almost impossible event). Contrast this with some actual temperature trajectories shown in Figure 5(a) for a very hot and a very cold day.

The Attributable Fraction (AF) was proposed (Steenland and Armstrong 2006; Gasparrini and Leone 2014), to interpret the risk estimates in terms of the observed data. If $R_{0}$ is the CR at the OT, then it can be interpreted as the risk of the “least-exposed” population. If $R_{1}$ is the CR at any other temperature value ($R_{1}>R_{0}$ by definition), we can define $\text{AF}=1-(R_{0}/R_{1})$ as the fraction of mortality cases attributable to temperature being different to the OT. For *observed temperature* $x_{t}$ on day *t*, the AF is

(16) $$\text{AF}(x_{t})=1-\frac{\;\text{exp}\;\left\{\sum_{l=0}^{L}h(l,\text{OT})\right\}}{\;\text{exp}\;\left\{\sum_{l=0}^{L}h(l,x_{t})\right\}}$$

$$=1-\;\text{exp}\;\left\{\sum_{l=0}^{L}\left[h(l,x_{t})-h(l,\text{OT})\right]\right\}.$$

Note the use of $x_{t}$ rather than $x_{t-l}$ reflecting how the risk of experiencing $x_{t}$ “today” is distributed over the next *L* days. This was termed as the forward AF in Gasparrini and Leone (2014). Then, the attributable number, $\text{AN}(x_{t})=\text{AF}(x_{t})\cdot {\tilde{y}}_{t}$, is the number of cases attributable to $x_{t}$, where ${\tilde{y}}_{t}$ is the mean mortality count in the period [t,t+L]. Finally, an overall estimate of the AF is obtained via $\bar{\text{AF}}=\sum_{t}\text{AN}(x_{t})/\sum_{t}y_{t}$ and here, this was estimated as $\bar{\text{AF}}=0.12\;[0.10,0.14]$. So 12% of deaths in Chicago can be attributed to non-optimal temperature values.

The AF can be computed for extremely high/low temperatures, termed heat-related AF (HAF) or cold-related AF (CAF). For temperature $>25.8^{{}^{\circ}}$C (95th quantile) the HAF was 0.11 [0.07, 0.15], while for temperature $<-7.8^{{}^{\circ}}$C (5th quantile) it was 0.27 [0.24, 0.30].

## 4. Interactions and Spatial Variability

### 4.1. Multiple Lagged Covariates

It is straightforward to include joint lagged covariate effects. Model2 was expanded to include the effect of PM10, a measure of air quality (particle size). The new model3 is

(17) $$\;\text{log}\;(\mu_{t})=\alpha_{\omega (t)}+\sum_{l=0}^{L}h(l,x_{t-l},z_{t-l})+\gamma_{\text{dow}(t)}$$

$$+f_{1}(\text{doy}(t))+f_{2}(\text{year}(t))$$

where *z* stands for PM10 and the function *h* is coded via:

te(TEMP,LAG,PM10,k = c(5,5,5),

bs = c("tp","tp","tp"))

We use 5 coefficients per dimension to keep computation low (k.check confirms adequacy). Model3 has AIC = 22464, much lower than model2 indicating better predictive performance. The estimated temperature-lag RR surface for 6 PM10 levels is given in the supplementary material. The plot indicates that PM10 modulates the temperature effect, but the weight of evidence is weak. A reason may be that PM10 is a short-lived pollutant and it is unlikely to have an effect up to 21 days, 5 days being more reasonable (Parliari et al. 2024). In mgcv we can construct tensor product interactions in way that separate the marginal from the joint effects using ti() rather than te(). This allows different lag-length per covariate and can be used to assess the significance of the interactions. We fit model4:

(18) $$\begin{array}{cc} \;\text{log}\;(\mu_{t}) & =\alpha_{\omega (t)}+\sum_{l=0}^{20}h_{1}(l,x_{t-l})+\sum_{l=0}^{4}h_{2}(l,z_{t-l}) \end{array}$$

$$\begin{array}{c} +\sum_{l=0}^{4}h_{3}(l,x_{t-l},z_{t-l})+\gamma_{\text{dow}(t)}\\ +f_{1}(\text{doy}(t))+f_{2}(\text{year}(t)) \end{array}$$

where the lagged effects are coded via:

ti(TEMP,LAG_TEMP,k = c(5,5))

+ ti(PM10,LAG_PM10,k = c(5,4)) + ti(TEMP_SHORT,PM10,LAG_PM10,k = c(5,5,4)), and where $h_{1}$ and $h_{2}$ are the marginal lagged effects of *x* and *z*. Function $h_{3}$ is constrained so that the marginal effects are excluded (a pure interaction term). A k.check indicates that the number of coefficients is adequate for each term and the summary of the model: summary(model4)

Approximate significance of smooth terms:

**Table:** 

	edf	Ref.df	Chi.sq	p-value
ti(TEMP,LAG_TEMP)	9.454	11.857	55.216	<2e-16***
ti(PM10,LAG_PM10)	1.311	1.532	0.175	0.843
ti(TEMP_SHORT,PM10,LAG_PM10)	5.956	8.345	11.948	0.177

indicates that the marginal PM10 effect is weak and so is its interaction with temperature. Note that model4 has a higher AIC (=28991) than model3 but we prefer it as it includes more physical understanding and the estimates are much more interpretable.

**Figure 6:**
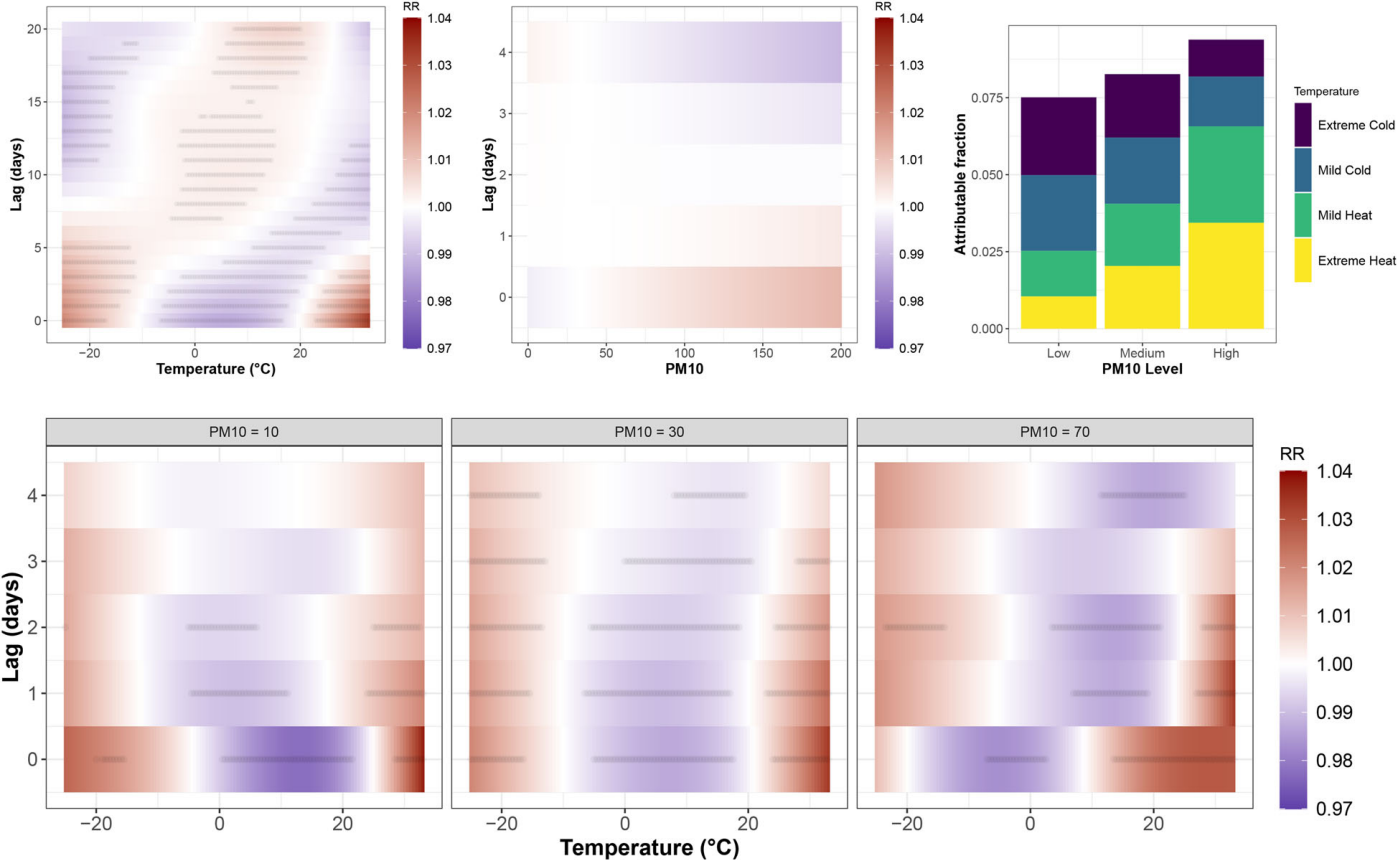
Estimates from model4. The marginal RR from temperature and PM10 are shown on the top two left plots, while the interaction term is given below (as temperature-lag surfaces for three different levels of PM10 (5%, 50%, and 95% sample quantiles)). Top right: AF for different levels of temperature and PM10 (temperature thresholds defined by the 0.05,0.25,0.75,0.95 sample quantiles and PM10 thresholds by 0.25 and 0.75.)

Figure 6 shows the RR estimates from model4. The marginal effect of temperature is similar the one from model2, while the PM10 marginal effect is evidently weak. The interaction is more interesting with clear indication that the surface changes across PM10 levels. The interaction is even more noticeable in the AF plot (Figure 6) where a much higher fraction of deaths is attributed to hot and polluted days.

### 4.2. Interactions with Temporal Covariates

It can be argued that temperature may have a different effect across time (e.g., seasons). To illustrate we allow the temperature-lag effect of model2 (not model4, for simplicity) to change with day-of-year. This is straightforward by considering the following model:

(19) $$\;\text{log}\;(\mu_{t})=\alpha_{\omega (t)}+\sum_{l=0}^{L}h(l,x_{t-l})+\sum_{l=0}^{L}h(l,x_{t-l},\text{DoY}(t))$$

$$+\beta_{\text{year}(t)}+\gamma_{\text{dow}(t)}+f_{\text{year}(t}(\text{doy}(t))$$

fitted via: model5 < - gam(Response ∼ omega

+ te(TEMP,LAG,k = c(7,7),bs = c("tp","tp")) + ti(TEMP,LAG,DOY,k = c(7,7,10), bs = c("tp","tp","cc")) + fYear +

s(DoY,bs="cc",k = 30,by = fYear)

+ s(DoW,bs="re"), family = gfam(list(nb,nb)),

method="REML",knots = list(DoY = c(0,366)))

where te(TEMP,LAG) is the marginal effect, and ti(TEMP,LAG,DOY is the interaction with day-of-year (DoY). The marginal effect of DoY is s(DoY,by = fYear), so each year has its own seasonal cycle. A summary of model5 indicates significant seasonal interaction while k.check confirms enough coefficients. The AIC of 34800 is slightly lower than model2 so the effect is small. This is confirmed by plots of the RR estimates for a day in each of the four seasons (see supplementary plots) which reveal small but intuitive differences, for example less pronounced heat-risk in autumn and winter days.

### 4.3. Smooth Spatial Variability

**Figure 7:**
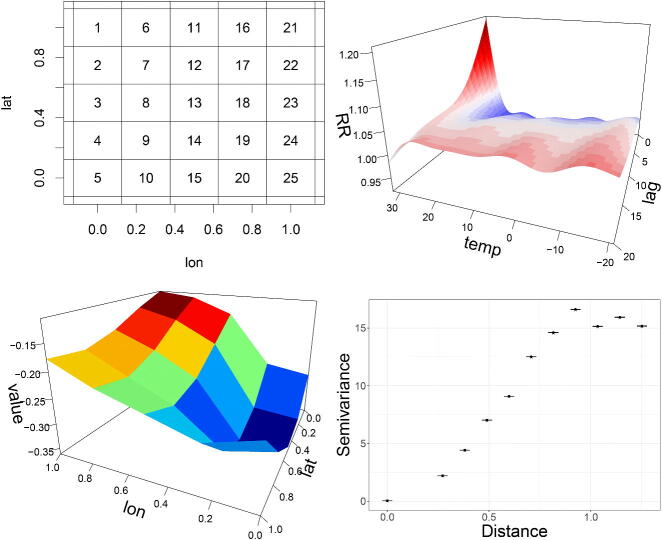
Left: Estimated RR surface from a simple NB Model fitted to the Chicago data. Middle: Smooth surface over fictitious coordinates. Right: Variogram of the simulated response values along with the variogram values of predictions (crosses relate to “observations”, points to predictions and bars to 95% prediction intervals, however, the intervals are tight and there is overlap of the crosses and points).

We simulate a situation where the temperature-lag relationship varies smoothly over a 5×5 grid (Figure 7 top left). The details are in the associated code and the 25 temperature-lag surfaces are given in the supplementary plots. In short, the coefficients of an estimated RR surface shown in Figure 7 (top right) are scaled using the surface in Figure 7 (bottom left). This results in 25 RR surfaces that vary smoothly across grid cells. For each, Poisson counts were simulated using the Chicago temperatures. Each grid cell is given a fictitious coordinate (lon, lat) and we fit a model with a spatially smooth temperature-lag effect:

(20) $$y_{s,t}∼\text{Poisson}(\mu_{s,t})\;\;t=\text{day and }s=\text{grid cell}$$

(21) $$\;\text{log}\;(\mu_{s,t})=\alpha +f(\text{lon}(s),\text{lat}(s))+\sum_{l=0}^{L}h_{1}(l,x_{t-l})$$

$$+\sum_{l=0}^{L}h_{2}(l,x_{t-l},\text{lon}(s),\text{lat}(s))$$

which is fitted via: modelS < - gam(yHere∼s(lon,lat,k = 20,

bs="tp")+te(TEMP,LAG,bs="tp",k = 7) + ti(TEMP,LAG,LON,LAT,bs="tp",

d = c(1,1,2),k = c(7,7,7)), family = poisson)

The term s(lon,lat) is a spatially isotropic smooth surface (same smoothness in all directions) using TPRS (Wood 2017). This is akin to an isotropic stochastic process, and is generally appropriate for covariates with the same units.

The argument d = c(1,1,2) gives an interaction between 3 splines: 1 for temperature, 1 for the lag and 1 for an isotropic TPRS of the coordinates. The estimated RR surfaces (supplementary plots) closely match the “true” values and we check spatial correlation using the variogram. Commonly used in geostatistics (Bailey and Gatrell 1995), this is the variance of the difference in values for any two locations, plotted against the distance between them. The smaller the variance is, the higher the correlation. Figure 7 (bottom right) shows the variogram for the simulated counts and corresponding predictions, indicating that spatial dependence is well captured (isotropic smoothing is adequate).

Alternatively, spatial structure can be constructed via a Markov Random Field (MRF) (Rue and Held 2005). A neighborhood structure for the grid cells is chosen a-priori and a Gaussian random effect is used, whose mean is a weighted sum of its neighbors. MRF-based effects are appropriate for gridded data or data over irregular areas (contiguous regions).

In mgcv, an MRF is constructed as a smooth function with a specific penalty configuration. We choose a next-nearest-grid-cell neighborhood structure and fit the following model:

(22) $$\;\text{log}\;\mu_{s,t}=\alpha +f(s)+\sum_{l=0}^{L}h_{1}(l,x_{t-l})+\sum_{l=0}^{L}h_{2}(l,x_{t-l},s)$$

where f(s) is an MRF spline, and where $h_{2}$ is a tensor product between temperature, lag and another MRF spline. The model is fitted via:

model_MRF < - gam(y∼s(fID,xt = list

(nb = neigh),bs="mrf") +

te(TEMP,LAG,bs="tp"),k = 10) +

ti(TEMP,LAG,IDmat,bs = c("tp","tp","mrf"),

k = c(10,10,25),

xt = list(nb = neigh)),family = poisson)

The argument bs="mrf" indicates an MRF spline and fID is a unique factor ID for each grid cell. The argument xt is a list which needs to contain the object nb that defines the neighboring structure. This object is a named list which has as many elements as there are spatial units (25 grid cells in this case). Each element of the nb list is a vector of the IDs of the grid cells that are neighbors to the one that the element relates to. For instance, grid cell 13 (see top left plot in Figure 7), would be an element of nb called “13”, and it would be the character vector (“7”,“8”,“9”,“12”,“14”,“17”,“18”,“19”) of its 8 neighbors (recall we chose a next-nearest-grid-cell neighborhood structure).

The model gives very similar RR estimates (see supplementary material) to modelS, and a virtually identical variogram check. However, the AIC of modelMRF is considerably lower. The bias and variability plots of the estimates (supplementary material), indicate slight under-estimation bias from modelS and more variability compared to model MRF. The MRF structure is thus superior at capturing smooth spatial variability here.

### 4.4. Hierarchical Structures

Often, the spatial structure of health data is provided on irregular shapes determined by administrative units. The assumption of spatial proximity (for an MRF) or smoothness in space (for isotropic smoothing) may be invalid, even when allowing for unit-specific covariates. Nevertheless there may be similarities in risk behavior across these units, which should be captured. This is important for pooling data across the units, for instance where in some units the data is scarce or where counts are low.

A way of achieving such pooling is to assume the categorical units are exchangeable, and construct a hierarchical structure with a “global” term plus unit-specific deviations. This is a hierarchical GAM (Pedersen et al. 2019):

(23) $$\;\text{log}\;(\mu_{t,s})=\alpha +\sum_{l=0}^{L}h(l,x_{t-l})+\sum_{l=0}^{L}h_{s}(l,x_{t-l})$$

$$=\alpha +f(x_{t})+f_{s}(x_{t})$$

where $f(x_{t})$ is the global or overall mean temperature-lag effect across all units *s*, and $f_{s}(x_{t})$ are the unit-specific deviations from $f(x_{t})$, so $\left(f(x_{t})+f_{s}(x_{t})\right)$ are the unit-specific effects. If there is little difference across units *s*, $f_{s}(x_{t})$ will be simpler than $f(x_{t})$ due to penalization, and therefore optimal use of the data is achieved (Gelman et al. 2013).

The term s(unit,bs="re") in mgcv gives a spline with a ridge penalty that emulates a $N(0,\sigma^{2})$ random effect, where the default number of coefficients is the number of units (Wood, Scheipl, and Faraway 2013; Wood 2016, 2017). Pooling the $h_{s}(l,x_{t-l})$ is achieved by constructing them as tensor products of lag *l*, covariate(s) $x_{t-l}$ and a random effect spline for *s*. The random effects induce shrinkage of $\left(f(x_{t})+f_{s}(x_{t})\right)$ toward $f(x_{t})$. We assess pooling by checking the between-unit correlation in posterior predictive simulations.

**Figure 8:**
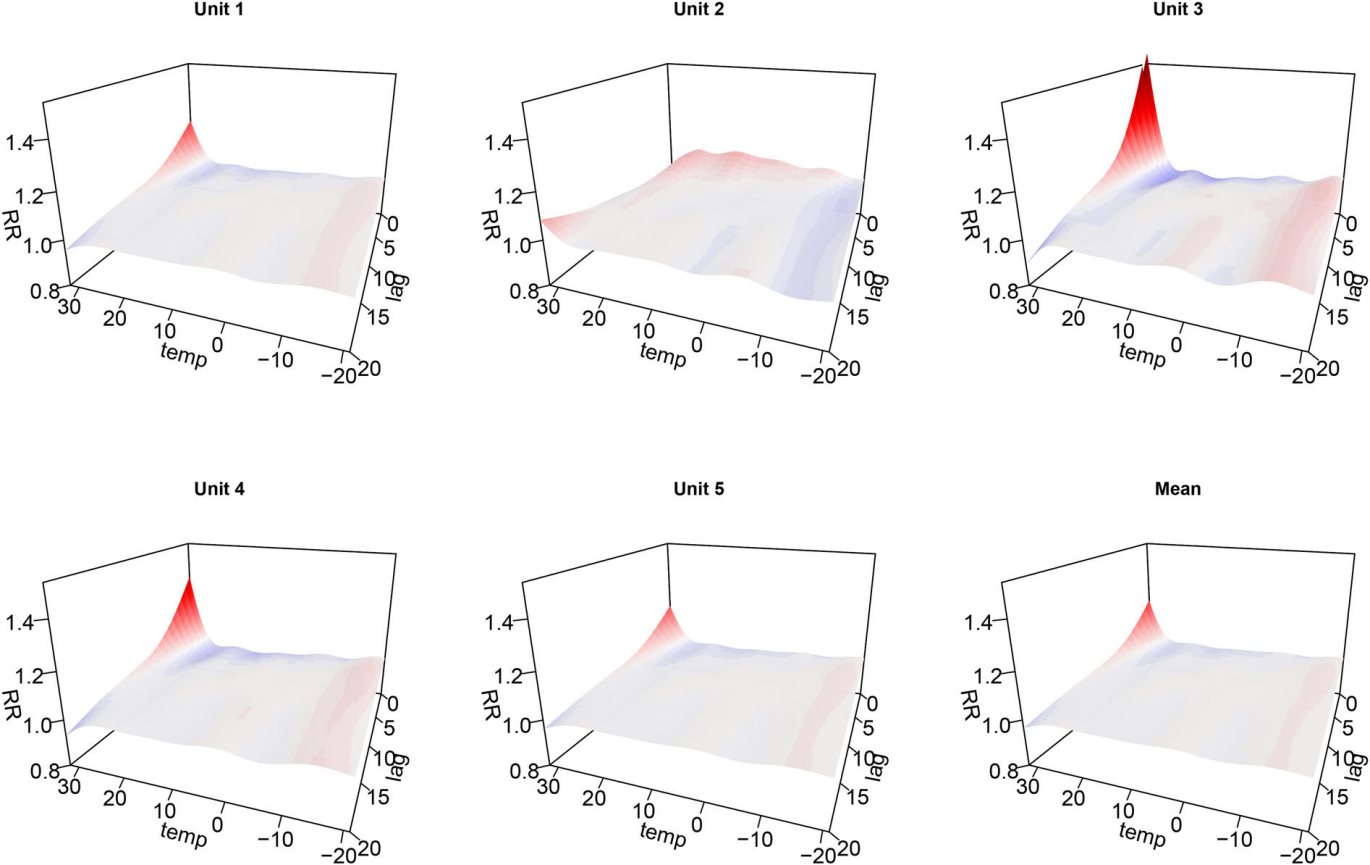
RR surfaces for 5 fictitious units based. The bottom right plot is the mean of the other 5.

We illustrate using simulation. The RR surface in Figure 7 (top right) was used to construct the 5 RR surfaces shown in Figure 8. Moreover, a seasonal (cyclic) trend plus a temporal trend were simulated for each of the artificial “units”, taking the form of smooth functions (shown in the supplementary material). This is done to assess the ability of the model to deal with temporal confounding in each unit. Poisson counts were simulated for each unit using the Chicago temperatures. The following model was then fitted:

(24) $$y_{s,t}∼\text{Poisson}(\mu_{s,t})\;\;t=\text{day and }s=\text{unit}$$

(25) $$\;\text{log}\;\mu_{s,t}=\alpha +\sum_{l=0}^{L}h_{1}(l,x_{t-l})+\sum_{l=0}^{L}h_{2}(l,x_{t-l},s)$$

$$+f_{s}(\text{doy}(t))+g_{s}(t)$$

where $f_{s}$ and $g_{s}$ are smooth functions of day-of-year and the daily time step respectively (one for each *s*) and where $h_{1}$ is the overall temperature-lag effect and $h_{2}$ are the unit-specific deviations. The model is fitted to the simulated data via:

pooling_model < - gam(y ∼ te(TEMP,LAG,

bs = c("tp","tp"),k = c(7,7)) +

t2(TEMP,LAG,UNIT,bs = c("tp","tp","re"),

k = c(10,10,5),m = 1,full = T) +

s(DoY,unit,bs="fs",k = 10,

xt = list(bs="cc")) + s(timeStep,

unit,bs="fs",k = 20),

family = poisson,method="REML")

The term t2(TEMP,LAG,UNIT) is $h_{2}(l,x_{t-l},s)$. The use of the t2 tensor (with argument full = T) is recommended for constructing random-effect deviations (Pedersen et al. 2019). (The t2 is not zero-centered for each unit so this is done manually in the online code.) The argument k = c(10,10,5) gives 10 coefficients per dimension plus 5 for the units. No other option than 5 is possible for random effect smooths, but here this is set explicitly in order to choose the other two. The deviations are given a higher number of coefficients than the global term (k = c(7,7)) to a-priori allow much flexibility in the deviations. The argument m = 1 imposes a first-order penalty on the deviations, to penalize the first rather than the second derivative. This gives smoother estimates and increases identifiability between $h_{1}$ and $h_{2}$. The two temporal trend terms use the fs basis, an alternative way to construct one smooth per unit (earlier we used the by argument).

The estimated surfaces are given in the supplementary material indicate that the model can indeed capture the simulated scenario. The bottom right plot is the estimate of $h(l,x_{t-l})$, the overall term, which closely resembles the mean of the true surfaces (bottom right of Figure 8).

In addition to the Bayesian Q-Q plot (supplementary material) we assess the ability of the model to pool the estimates, as quantified by the correlation of the counts across the 5 units. Table 2 compares true and predicted correlations indicating that between-unit correlation is well captured.

**Table 2:** Sample and estimated correlations of the simulated counts for each fictitious unit.

	Unit 1	Unit 2	Unit 3	Unit 4	Unit 5
Unit 1	1.00	−0.91	0.73	0.70	0.72
Unit 2	−0.90 [−0.92, −0.91]	1.00	−0.77	−0.75	−0.74
Unit 3	0.73 [0.71, 0.74]	−0.77 [−0.78, −0.76]	1.00	0.89	0.66
Unit 4	0.70 [0.68, 0.72]	−0.75 [−0.75, −0.74]	0.89 [0.89, 0.90]	1.00	0.40
Unit 5	0.72 [0.70,0.74]	−0.74 [−0.75, −0.73]	0.66 [0.65, 0.67]	0.40 [0.39,0.42]	1.00

NOTE: The top diagonal entries are the sample cross-correlations across the 5 units. The bottom diagonal entries are corresponding correlations of 1000 simulations from the posterior predictive distribution of the counts for each unit. The values shown are the posterior mean and the 95% prediction intervals.

Lastly, the bias and variability in the estimates are shown in the supplementary material. The estimates are unbiased, however the differences become larger at very high temperatures across all 5 units (but generally range between –0.05 and 0.05). Moreover, the variability is 0.05 on average with no distinguishable pattern across temperature quantiles, except for the highest quantile, where more extremes are evident.

## 5. Computation

All models were fit on a laptop using Ubuntu 22.04 with 16GB RAM and an 8-core Intel Core i7-8550U CPU at 1.80GHz. The OpenBLAS library was used, enabling parallelization of matrix calculations when using gam(). Some of the models presented can be computationally intensive (generally computation depends on number of data points and/or coefficients).

Expensive GAMs will require a lot of RAM and can benefit from parallel computation. Function bam() (Wood, Goude, and Shaw 2015) in mgcv reduces RAM usage and enables parallelization. The online supplementary material (Economou 2025) includes a mini-tutorial for implementing the pooling model (24)–(25) with parallel computation using two different ways. Table 3 shows computation times using the standard gam() function with OpenBLAS enabled and also with the two bam() options.

**Table 3:** Computation times in seconds for each of the models presented (columns).

Model	1	2	3	4	5	S	MRF	pooling
gam()	6	97	79	140	704	1871	1124	202
bam() with parallel	24	NA	NA	NA	NA	110	96	60
bam() with nthreads	13	72	73	50	205	112	168	38

NOTE: Row 1: gam() with OpenBLAS library, 2: bam() using the parallel package and 3: bam() using the nthreads option. The NAs relate to the fact that currently using family = gfam produces an error when using the parallel approach.

## 6. Summary and Conclusions

The aim of this tutorial was to illustrate the modeling flexibility offered by mgcv for fitting DLNMs. As highlighted in the literature (Gasparrini et al. 2017), implicit penalization in GAMs ensures optimal estimates of the lagged effects (for a given maximum lag), which is important for robust risk estimation in epidemiological data. A wide range of modeling scenarios was covered: data with temporal and spatial structure; allowing for outliers via mixture distributions; interaction of the lagged effects with other covariates including time and space; and hierarchical formulations for data pooling across categorical units. The latter constitutes a novelty in the DLNM literature.

The work presented was inspired by and built upon many years of research on using DLNMs across many areas (e.g., Zanobetti 2000; Gasparrini, Armstrong, and Kenward 2010; Gasparrini and Leone 2014; Obermeier et al. 2015). The work here provides a practically useful extension of the DLNM framework, particularly in the area of epidemiology, and *in our opinion* provides a unifying approach that allows for penalization of the smooth functions involved, pooling of the data, interactions between multiple covariates and Bayesian inference.

One thing we did not explicitly illustrate is space-time interaction, which however is trivial given the tools presented. Tensor product interactions between time and space quantities can be constructed as described in Sections 4.2–4.4.

As with any modeling tool, there are some caveats to be addressed in future work. On the theoretical side, the Bayesian inference presented is conditional on both the penalty parameters and any hyperparameters of the conditional distribution (e.g., the dispersion parameter of the Negative Binomial). In our experience the uncertainty from these is relatively small but it is a potential issue when claiming to fully quantify uncertainty. On the practical side, computation efficiency can be an issue when fitting GAMs in mgcv, particularly for a large number of coefficients in the splines. There are options in mgcv to deal with the “many-coefficients” and/or the “many-covariates” situation (e.g., function bam and parallel computing), demonstrated in the online supplementary material.

## Supplementary Materials

All work presented in this article is completely reproducible via the online supplementary material, which includes data, code, instructions and supplementary figures. This can be accessed at Zenodo (Economou 2025). All models, estimates and model checking presented are reproducible, although some deviation may be present due to differences in random number generation across machines and/or R versions. For this work we used R (R Core Team 2024) version 4.4.2 and mgcv version 1.9-1.
